# Ethnobotany, phytochemistry and pharmacological properties of Fagopyri Dibotryis Rhizoma: A review

**DOI:** 10.3389/fphar.2023.1095554

**Published:** 2023-03-06

**Authors:** Qi Geng, Bin Liu, Zhiwen Cao, Li Li, Peipei Lu, Lin Lin, Lan Yan, Cheng Lu

**Affiliations:** Institute of Basic Research in Clinical Medicine, China Academy of Chinese Medical Sciences, Beijing, China

**Keywords:** Fagopyri Dibotryis Rhizoma, ethnobotany, application, phytochemistry, pharmacology

## Abstract

*Fagopyri Dibotryis Rhizoma* (FDR) is an effective Chinese herbal medicine with a long history of use in China. FDR is effective in heat clearing and detoxifying, promotion of blood circulation, relieving carbuncles, dispelling wind, and removing dampness. Its seeds also have high nutritional value, are rich in protein, and contain a variety of mineral elements and vitamins. Therefore, FDR is considered a natural product with medical and economic benefits, and its chemical composition and pharmacological activity are of interest to scientists. The current review provides an overview of the available scientific information on FDR, particularly its botany, chemical constituents, and pharmacological activities. Various sources of valid and comprehensive relevant information were consulted, including the China National Knowledge Infrastructure, Web of Science, and PubMed. Among the keywords used were “*Fagopyri Dibotryis Rhizoma*”, “botanical features”, “chemical composition”, and “pharmacological activity” in combination. Various ailments are treated with FDR, such as diabetes, tumor, sore throat, headache, indigestion, abdominal distension, dysentery, boils, carbuncles, and rheumatism. FDR is rich in organic acids, tannins, flavonoids, steroids, and triterpenoids. Experiments performed *in vitro* and *in vivo* showed that FDR extracts or fractions had a wide range of pharmacological activities, including antitumor, anti-inflammatory, immunomodulatory, antioxidant, antimicrobial, and antidiabetic. The current review provides an integrative perspective on the botany, phytochemistry and pharmacological activities of FDR. FDR may be used as a medicine and food. Based on its chemical composition and pharmacological effects, the main active ingredients of FDR are organic acids, tannins, and flavonoids, and it has obvious antitumor pharmacological activity against a variety of malignant tumors. Therefore, FDR is worthy of further study and application as a potential antitumor drug.

## 1 Introduction

Chinese herbal medicine plays a crucial role in the prevention and treatment of diseases as a drug resource for the traditional medical system and as an important raw material for chemical drugs, international botanicals, and the food industry. A significant amount of evidence suggests that medicinal plants may be used to treat a variety of diseases and for the discovery of novel pharmacologically active molecules. The phytochemicals identified from medicinal plants have provided promising lead compounds for effective new drugs (Ríos and Recio, 2005; Batiha et al., 2019a; Batiha et al., 2020; El-Saber et al., 2020). Medicinal plants have gained wider acceptance in recent years due to the perception that they are natural products and less likely to induce side effects than their synthetic counterparts (Abushouk et al., 2017a; Abushouk et al., 2017b). Various medicinal plants possess anti-inflammatory, antibacterial, antitumor, antiviral, and other activities (Bakkali et al., 2008). Herbal extracts and pharmacologically active molecules extracted from different plant species that were previously used in traditional medicine have received much attention (Essawi and Srour, 2000; Batiha et al., 2019b; Beshbishy et al., 2019).

*Fagopyrum dibotrys (D. Don) Hara* is a perennial herb of the genus *Fagopyrum* in the family *Polygonaceae*, and it is widely distributed in the Sichuan Basin, the hills of Guangdong and Guangxi, and the Yunnan-Guizhou Plateau in China and Thailand, Nepal, India and other countries (Peng et al., 1996). The dried rhizome is often used as medicine and food because it effectively clears heat, removes toxins, drains pus, removes blood stasis and invigorates the spleen to strengthen the stomach. For several thousand years in China, *Fagopyri Dibotryis Rhizoma* (FDR) has been widely used as a folk medicine to cure forms of chronic bronchitis, lung cancer, sore throat, rheumatic disease, dysentery, and enteritis (Chan, 2003a; Jing et al., 2016; Zhao et al., 2018). The medicinal properties of FDR are attracting the attention of an increasing number of academics due to its tremendous medicinal value. FDR components have been extensively examined, and an increasing number of compounds have been identified and isolated. A variety of components have been identified in FDR, including organic acids, tannins, flavonoids, steroids, and triterpenoids (Shao et al., 2005; Cao et al., 2019). FDR also has a broad spectrum of pharmacological effects, including antitumor, antimicrobial, anti-inflammatory, antioxidant, and immunomodulatory effects (Chan, 2003b; Shen, 2013; Wang et al., 2017).

FDR has a variety of chemical components and diverse pharmacological activities, and it is a highly valuable medicinal resource plant for development. Many studies recently investigated the botany, phytochemistry and pharmacology of FDR and found that organic acids, tannins, and flavonoids were the most important active components underlying the broad-spectrum antitumor, anti-inflammatory, and other effects (Li et al., 2019). However, comprehensive and up-to-date information on FDR is lacking. Therefore, the current review summarizes recent progress on the phytoconstituents, chemical components and pharmacological activity of FDR, especially the organic acids, tannins and flavonoids that inhibit tumors and the specific mechanisms of these effects, and adds its botanical characteristics and clinical applications. Various published data of valid and comprehensive relevant information were consulted, including the China National Knowledge Infrastructure, Web of Science, and PubMed. Among the keywords used were “*Fagopyri Dibotryis Rhizoma*”, “botanical features”, “chemical composition”, and “pharmacological activity” in combination. This review provides references for the further development and use of FDR in traditional Chinese medicine.

## 2 Botanical characterization and application

### 2.1 Botanical characterization

*Fagopyrum dibotrys (D. Don) Hara* is a perennial herb that is native to eastern, central and southwestern China, India, Nepal, Vietnam, Thailand, and other countries. The habitat of *Fagopyrum dibotrys (D. Don) Hara* is 250–3,200 m above sea level in valley wetlands and hillside forests. The rhizomes are black‒brown, stout, and ligneous, and the stems are long and erect, green, or brownish, 40–100 cm high, branched, striate, and glabrous. The petiole is 2–10 cm, and the leaf blade is triangular at 4-12 × 3–11 cm. Both surfaces are papillate, the base is nearly hastate, the leaf margin is entire, and the apex is acuminate. The ocrea is brown, 5–10 mm, membranous, and oblique, and the apex is truncate, not ciliate. Plants have terminal, axillary or corymbose inflorescence. Bracts are ovate-lanceolate, ca. 3 mm, with membranous margins, and an acute apex, each 4-flowered and rarely 6-flowered. Pedicels are in equaling bracts that articulate at the middle. Perianth are white, and tepals are narrowly elliptical, ca. 2.5 mm. Stamens are included. The styles are free, and stigmas are much longer than the persistent perianth, capitate, and opaque. During April-August, the chenes are blackish brown, dull, broadly ovoid, 6–8 mm long, trigonous, sometimes narrowly winged, with smooth to repandous angles, and an acute apex (Editorial Committee of Chinese Flora, 1998).

### 2.2 Application

The anti-inflammatory and antiseptic effects of FDR may be used to treat a variety of respiratory diseases. FDR tablets combined with tiotropium bromide powder nebulizer exhibited clinical efficacy and high safety, and it effectively improved the acute exacerbation of COPD patients with clinical symptoms and blood gas analysis indicators and reduced the inflammatory response (Li et al., 2022). FDR capsules combined with salmeterol ticapone inhalation powder nebulizer for the treatment of bronchial asthma in children had good results, and it effectively relieved clinical symptoms, improved lung and immune functions, regulated serum inflammatory factor levels, and had a good safety profile (Wei et al., 2022). FDR capsules significantly reduced the acute exacerbation of asthma patients’ serum EOS and IgE levels, reduced the respiratory inflammatory response, improved the patient’s lung ventilation function and the clinical symptoms of patients, which are worthy of clinical promotion (Feng et al., 2021).

The anti-inflammatory, analgesic and antibacterial pharmacological effects of FDR significantly improved the symptoms of infectious diseases of the intestinal tract. FDR tablets combined with cefdinir dispersible tablets effectively improved the symptoms of acute bacterial dysentery patients with diarrhea, purulent stools and other symptoms and reduced the level of serum inflammatory indicators (Zhang and Li, 2019).

## 3 Phytochemistry

Various parts of FDR have yielded more than 100 compounds, including organic acids, tannins, flavonoids, steroids, and triterpenoids (Lin et al., 2016), which support its potential use as a medicinal and food plant. These compounds likely explain the differentiated pharmacological effects based on the characteristics of these chemical components. A list of phytochemical constituents is presented in Table 1.

**TABLE 1:** Phytochemical constituents of FDR.

No.	Chemical component	Plant Part	Chemistry	Chemical Formula	Chemical Structures	Biological activity
1	(-)-Epicatechin-3-O-gallate acid ester	Rhizome	Organic acids	C_22_H_18_O_10_	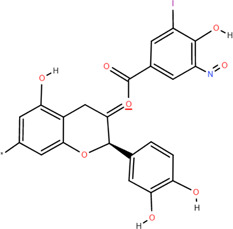	Anti-inflammatory Antioxidant
2	Gallic acid	Rhizome	Organic acids	C₇H₆O₅	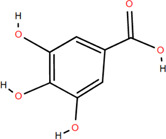	Antitumor
						Antimicrobial
						Antioxidant
3	Protocatechuic acid	Rhizome	Organic acids	C_7_H_6_O_4_	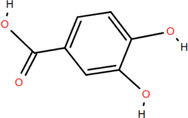	Anti-inflammatory
						Antimicrobial
4	3,4-Dihydroxy benzamide	Rhizome	Organic acids	C_7_H_7_O_3_	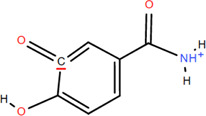	Anti-inflammatory
						Antimicrobial
5	Monopalmitin	Rhizome	Organic acids	C_19_H_38_O_4_	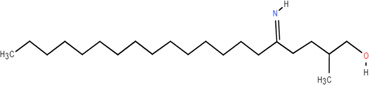	Immunomodulatory
6	Protocatechuic acid methyl ester	Rhizome	Organic acids	C_8_H_8_O_4_	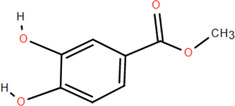	Antioxidant
7	Tans-p-hy-droxy cinnamic methyl ester	Rhizome	Organic acids	C_10_H_10_O_3_	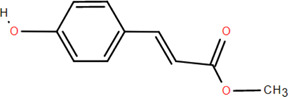	Antitumor
						Antimicrobial
8	3,5-Dimethoxy benzene carbonic acid-4-O-glucoside	Rhizome	Organic acids	C_12_H_18_O_3_	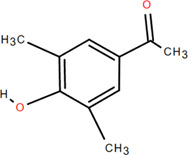	Anti-inflammatory
9	Ferulic acid	Rhizome	Organic acids	C_10_H_10_O_4_	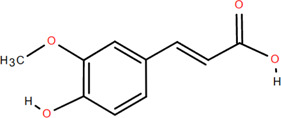	Antioxidant
						Antimicrobial
						Anti-viral
10	Syringic acid	Rhizome	Organic acids	C_9_H_10_O_5_	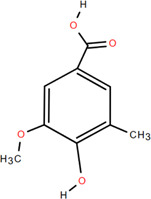	Antimicrobial
11	p-Hydroxyl-benzaldehyde	Rhizome	Organic acids	C_7_H_6_O_2_	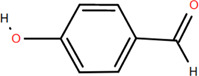	Anti-inflammatory
						Antimicrobial
12	Succinic acid	Rhizome	Organic acids	C_4_H_6_O_4_	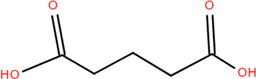	Antimicrobial
						Immunomodulatory
13	Luteolin	Rhizome	Flavonoids	C_15_H_10_O_6_	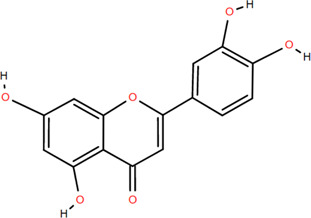	Anti-inflammatory
14	(-)-Epicatechin	Rhizome	Flavonoids	C_15_H_14_O_6_	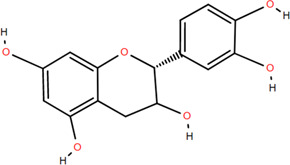	Antitumor
						Anti-inflammatory
15	3-Galloyl (-) epicatechin	Rhizome	Flavonoids	C_22_H_18_O_10_	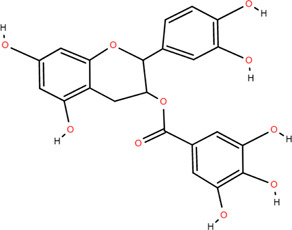	Antitumor
						Antioxidant
						Anti-inflammatory
16	Dimeric procyanidin	Rhizome	Flavonoids	C_45_H_38_O_18_	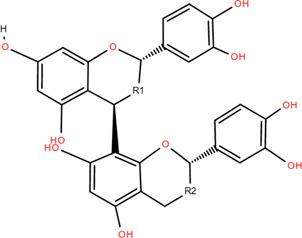	Anti-inflammatory
						Antimicrobial
						Immunomodulatory
17	(+)-Catechin	Rhizome	Flavonoids	C_15_H_14_O_6_	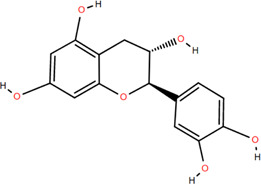	Antitumor
						Antidiabetic
						Anti-inflammatory
18	Eriodictyol	Roots	Flavonoids	C_15_H_12_O_6_	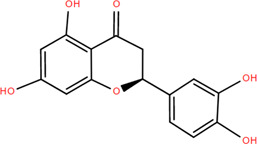	Anti-inflammatory
						Antioxidant
						Antidiabetic
19	Quercetin	Seeds, Stems, Roots, Leaves	Flavonoids	C_15_H_10_O_7_	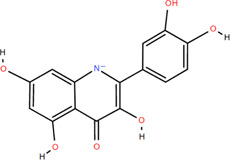	Anti-inflammatory
						Antioxidant
						Antimicrobial
						Immunomodulatory
20	Rutin	Flowers, Seeds, Leaves	Flavonoids	C_27_H_30_O_16_	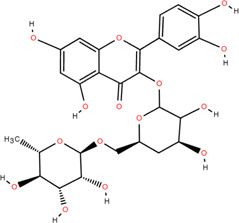	Anti-inflammatory
						Antioxidant
						Anti-viral
21	Genkwanin	Rhizome	Flavonoids	C_16_H_12_O_5_	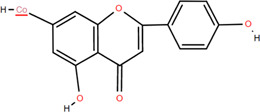	Antitumor
						Anti-viral
22	Chrysoeriol	Rhizome	Flavonoids	C_16_H_12_O_6_	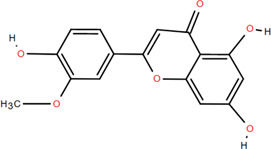	Anti-inflammatory
23	Pratol	Rhizome	Flavonoids	C_16_H_12_O_4_	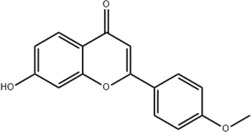	Anti-inflammatory
24	Luteolin-7,4′-dime-thylether	Rhizome	Flavonoids	C_17_H_14_O_6_	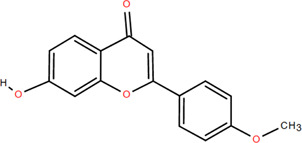	Anti-inflammatory
25	Rhamnetin	Rhizome	Flavonoids	C_16_H_12_O_7_	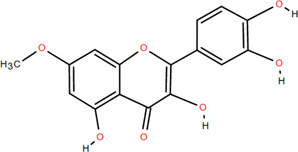	Antitumor
						Anti-inflammatory
26	3,6,3′,4′-Tetrahydroxy-7-methoxyflavon	Rhizome	Flavonoids	C_16_H_12_O_7_	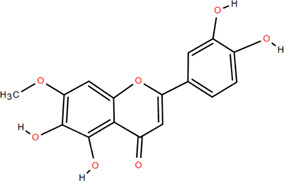	Anti-inflammatory
27	Procyanidin B2	Rhizome	Tannins	C_30_H_26_O_12_	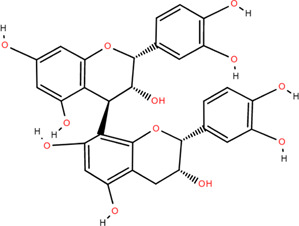	Anti-inflammatory Antimicrobial
28	Procyanidin C1	Rhizome	Tannins	C_45_H_38_O_18_	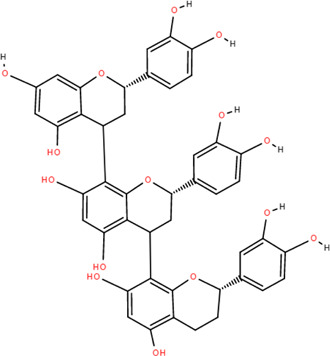	Antitumor
						Anti-diabetic
29	Procyanidin B4	Rhizome	Tannins	C_30_H_26_O_12_	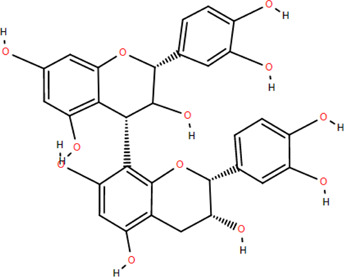	Antitumor
						Antioxidant
30	3,3'-Digalloyl procyanidin B2	Rhizome	Tannins	C_44_H_34_O_20_	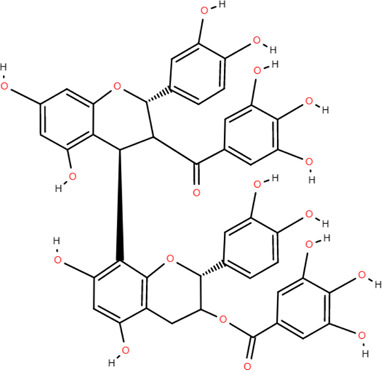	Antitumor
						Antioxidant
31	β -Sitosterol	Rhizome	Steroids	C_30_H_52_O	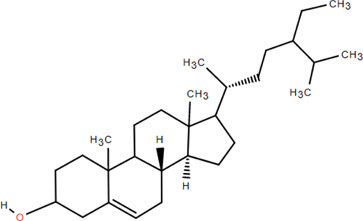	Antitumor
32	β -Daucosterol	Rhizome	Steroids	C_35_H_60_O_6_	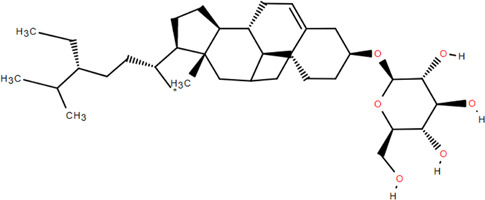	Antitumor
33	Hecogenin	Rhizome	Steroids	C_27_H_42_O_4_	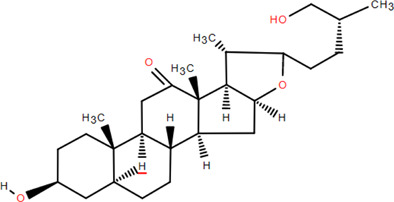	Anti-inflammatory
34	Glutinone	Rhizome	Terpenoids	C_19_H_18_O_3_	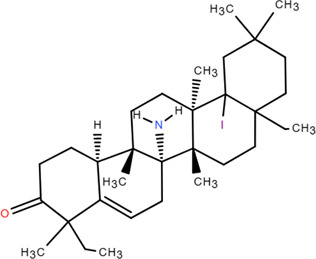	Antimicrobial
35	Glutinol	Rhizome	Terpenoids	C_30_H_50_O_1_	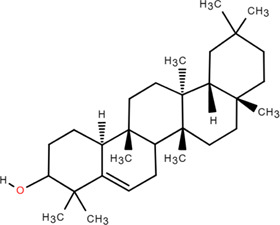	Antimicrobial
36	N-Butyl*- β -*D-fructopy-ronoside	Rhizome	Others	C_10_H_20_O_6_	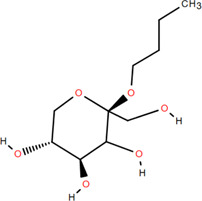	Antitumor
37	Methyl-3,4-dihydroxybenzoatem	Rhizome	Others	C_8_H_8_O_4_	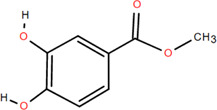	Anti-inflammatory
						Antioxidant
38	Gglycerol monop-almitate	Rhizome	Others	C_19_H_38_O_4_	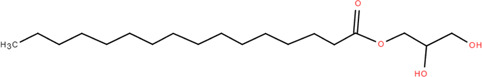	Anti-inflammatory
39	p-Hydroxyl-benzaidehyde	Roots	Others	C_7_H_6_O_2_	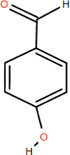	Anti-inflammatory
						Antimicrobial
40	N-Trans-coumaroyl tyramine	Rhizome	Others	C_17_H_17_NO_3_	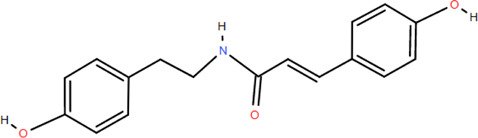	Anti-inflammatory
41	Emodin	Rhizome	Others	C_15_H_10_O_5_	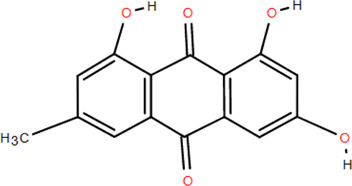	Antimicrobial
42	Diboside A	Rhizome	Others	C_49_H_48_O_20_	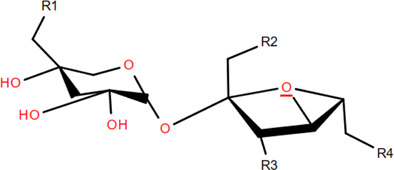	Anti-inflammatory
43	3-Methyl-gossypetin 8-O-d-glucopyranoside	Rhizome	Others	C_22_H_22_O_13_	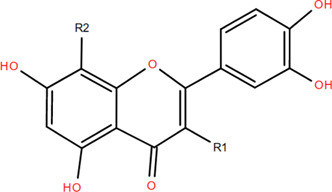	Anti-inflammatory
44	5,5-Di-*α*-furaldehyde dimethylether	Rhizome	Others	C_7_H_10_O_3_	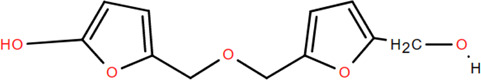	Immunomodulatory

### 3.1 Organic acids

Organic acids are compounds that contain -COOH, -SO_3_H, RSOOH, and RCOSH in their molecular structure, and leaves, roots, and Chinese herbs are abundant in these molecular structures. Twelve organic acids have been identified in FDR, including gallic acid, protocatechuic acid, (-)-epicatechin (Li et al., 2020; Huang et al., 2022), (-)-epicatechin-3-O-gallate acid ester, tans-p-hy-droxy cinnamic methyl ester, 3,4-dihydroxy benzamide, monopalmitin, protocatechuic acid methyl ester (Shao et al., 2004), 3,5-dimethoxy benzene carbonic acid-4-O-glucoside, syringic acid, ferulic acid, p-hydroxyl-benzaldehyde, and succinic acid (Zhao et al., 2011).

### 3.2 Flavonoids

Flavonoids are widely present in naturally growing plants and refer to a class of compounds with two benzene rings connected by three carbon atoms that create the C6-C3-C6 structure (Cook and Samman, 1996). Quercetin, rutin (Tang et al., 2014), luteolin (Shao et al., 2005), genkwanin, chrysoeriol (Yan, 2006), pratol, luteolin-7,4′-dime-thylether, rhamnetin, iorhamnetin, 3,6,3′,4′-tetrahydroxy-7-methoxyflavon (Zhang et al., 2016), eriodictyol (Zhao et al., 2011), dimeric procyanidin (Liu et al., 1983), 3-galloyl (+) catechin, 3-galloyl (-) epicatechin (Liu et al., 1998), (+)-catechin, (-) epicatechin (Zhang et al., 1994) and other flavonoids were isolated from FDR using column chromatography and high-performance liquid chromatography (HPLC).

### 3.3 Tannins

Tannins are phenolic compounds with complex structures that are widely distributed in plants. Procyanidin b2, procyanidin c1 (Huang et al., 2022), procyanidin b4 (Peng et al., 1996), and 3,3′-digalloyl procyanidin b2 were isolated from FDR.

### 3.4 Steroids

Steroids are a class of natural chemical components that exist widely in nature and have the steroid parent nucleus of cyclopentane-polyhydrophenanthrene in their structure. Chromatography on silica and Sephadex LH-20 columns isolated *β*-sitosterol and *β*-daucosterol from FDR (Wu et al., 2008). Liu et al*.* obtained hecogenin from FDR (Liu et al., 1983).

### 3.5 Terpenoids

Terpenoid is a general term that summarizes all polymers of isoprene and their derivatives, which are commonly found in plants. Terpenoids have important physiological activities and are an important resource for the study of natural products and the development of new drugs. Silica gel column chromatography, Sephadex LH-20 column chromatography and recrystallization were used to separate the ethyl acetate extract as glutinone and glutinol (Shao et al., 2005).

### 3.6 Other components

Emodin (Wu et al., 2008), glycerol monop-almitate, n-butyl-*β*-D-fructopy-ronoside, methyl-3,4-dihydroxybenzoate (Shao et al., 2005), diboside A, 3-methyl-gossypetin 8-O-d-glucopyranoside (Wang et al., 2005), 5,5-di-*α*-furaldehyde dimethylether (Tian et al., 1997), n-trans-coumaroyl tyramine, and p-hydroxy-benzaidehyde (Zhao et al., 2011) were also isolated from FDR.

## 4 Pharmacological activities

**FIGURE 1:**
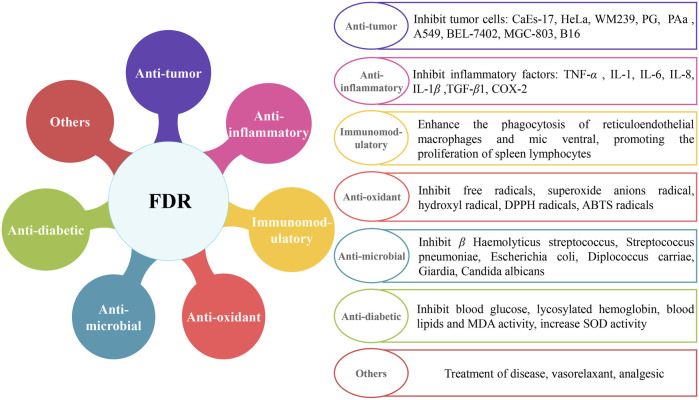
Molecular pharmacological activity mechanisms of FDR.

FDR is widely used in Chinese herbal medicine for its antitumor, anti-inflammatory, antimicrobial, antioxidant, and immunomodulatory properties in recent years (Figure 1). A variety of extracts and their chemical constituents showed various and significant biological and pharmacological activities in previous studies (Yang et al., 2019). Extracts and constituents of FDR were tested, and the results support their renowned applications in the treatment of a variety of ailments. Detailed pharmacological studies are discussed in the following sections.

### 4.1 Antitumor activity

**FIGURE 2:**
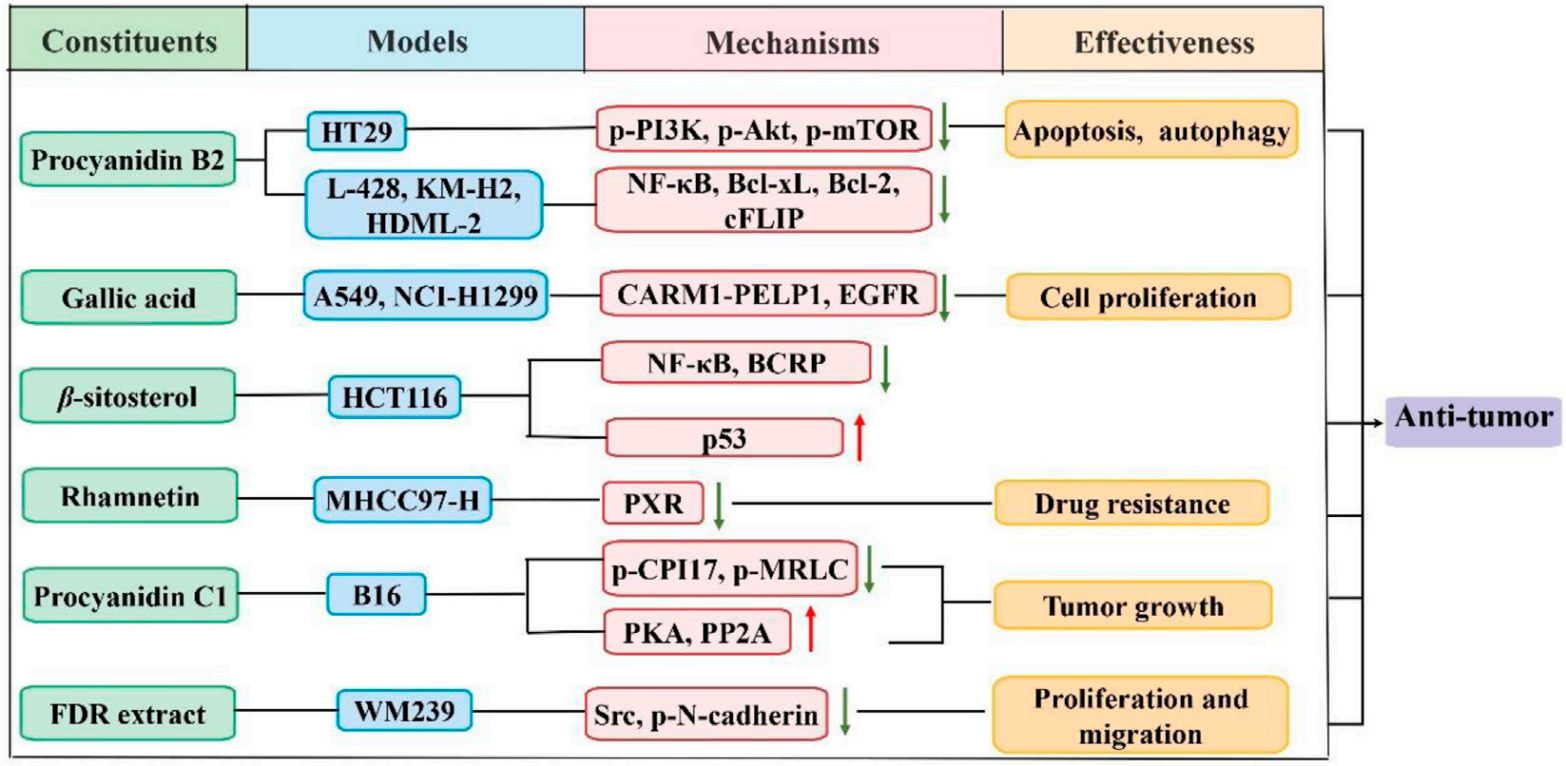
Schematic diagram of the antitumor mechanisms of FDR and its constituents.

The antitumor activity of FDR has drawn increasing attention over the past decades. FDR components had beneficial effects in the treatment of a variety of cancers in several studies. As shown in Figure 2, Wang et al. (Wang and Bao, 2020) found that gallic acid prevented non-small cell lung cancer progression *via* inhibition of epidermal growth factor receptor activation and impairment of the binding of coactivator-associated arginine methyltransferase 1 to proline, glutamic acid, and leucine-rich protein 1. Vergara et al. (Pereyra-Vergara et al., 2020) showed that reactive oxygen species (ROS) mediated (-)-epicatechin-induced apoptosis in human breast cancer cells. Apoptosis and autophagy were induced by procyanidin b2 in colorectal cancer cells (CRC) in a dose-dependent manner *via* downregulation of the expression of phosphorylated-phosphatidylinositol 3-kinase (p-PI3K), phosphorylated-protein kinase B (p-Akt) and phosphorylated-mammalian target of rapamycin (p-mTOR) of the PI3K/Akt pathway (Zhang et al., 2019). Procyanidin b2 prevented the binding of nuclear factor kappa B (NF-*κ*B) to DNA in the H-RS cell line and inhibited NF-*κ*B-driven genes, including anti-apoptotic proteins (Mackenzie et al., 2008). Another study revealed that *β*-sitosterol regulated the treatment response in CRC by mediating the p53/NF-*κ*B/BCRP signal transduction axis (Wang et al., 2020). Melanoma cell growth inhibition by procyanidin c1 was attributed to activation of the 67LR/PKA/PP2A/CPI17/MRLC pathways (Bae et al., 2020). Genkwanin increased host immunity and decreased the levels of inflammatory cytokines, which may make it an effective chemotherapeutic agent for the treatment of CRC (Wang X et al., 2015). Rhamnetin inhibited the expression of the pregnant x receptor (PXR) by increasing miR-148a levels, which decreased the expression of its downstream genes. Therefore, sorafenib was more effective against hepatocellular carcinoma (Li Y et al., 2021).

The treatment of cancer cells with FDR extract inhibited their growth. FDR extract showed significant proliferation inhibitory activity on HeLa cells, which was primarily associated with modulation of the expression of the apoptotic inducible factor Bcl2-associated X (Bax) and inhibition of the anti-apoptotic factor B-cell lymphoma-2 (Bcl2). The extract of FDR can also activated caspase-8, caspase-9, and caspase-3 and released mitochondrial cytochrome C (Pan et al., 2018). Some extracts from FDR possessed potential antitumor activity. For example, an extract from the FDR rhizome had antiproliferative and proapoptotic effects on the human esophageal cancer cell line CaEs-17 (Zhang et al., 2010). Chen et al. (Chen et al., 2012) revealed that FDR prevented Bowes melanoma cell WM239 proliferation and migration, which was accomplished *via* reduced activation of Src protein, decreased levels of N-cadherin intracellular segment phosphorylation and dissociation of N-cadherin from *β*-catenin. Fr4 is a polyphenolic substance extracted from FDR. Fr4 reduced tumor weight, increased tumor suppression, and showed good antitumor activity in a mouse Lewis lung cancer model (Chen et al., 2005). Fr4 promoted a dose-dependent increase in the inhibition of HL-60 proliferation in leukemic cells and induced apoptosis (Chen et al., 2006). The FDR extract Fr4 also had an antitumor effect on kidney cancer. Fr4 inhibited the proliferation and induced apoptosis of kidney cancer cells *via* a mechanism related to the upregulation of DNA damage-induced transcript 4 protein expression (Song et al., 2020).

Wei Mai Ning capsules are the main raw material extracts from FDR, which inhibit tumor growth, invasion, and blood flow metastasis, and it has been approved for clinical cancer therapy (Lou et al., 2004a). Wei Mai Ning had effects on the lung cancer cell lines PG, PAa and A549 and inhibited the liver cancer cell line BEL-7402, gastric cancer cell line MGC-803 and melanoma cell line B16 to varying degrees (Lou et al., 2004b). Wei Mai Ning inhibited the adhesion between PG and HUVECs *in vitro via* the dual action of PG cells and HUVECs, which inhibited tumor cell metastasis in the blood channel (Lou et al., 2007).

### 4.2 Anti-inflammatory activity

**FIGURE 3:**
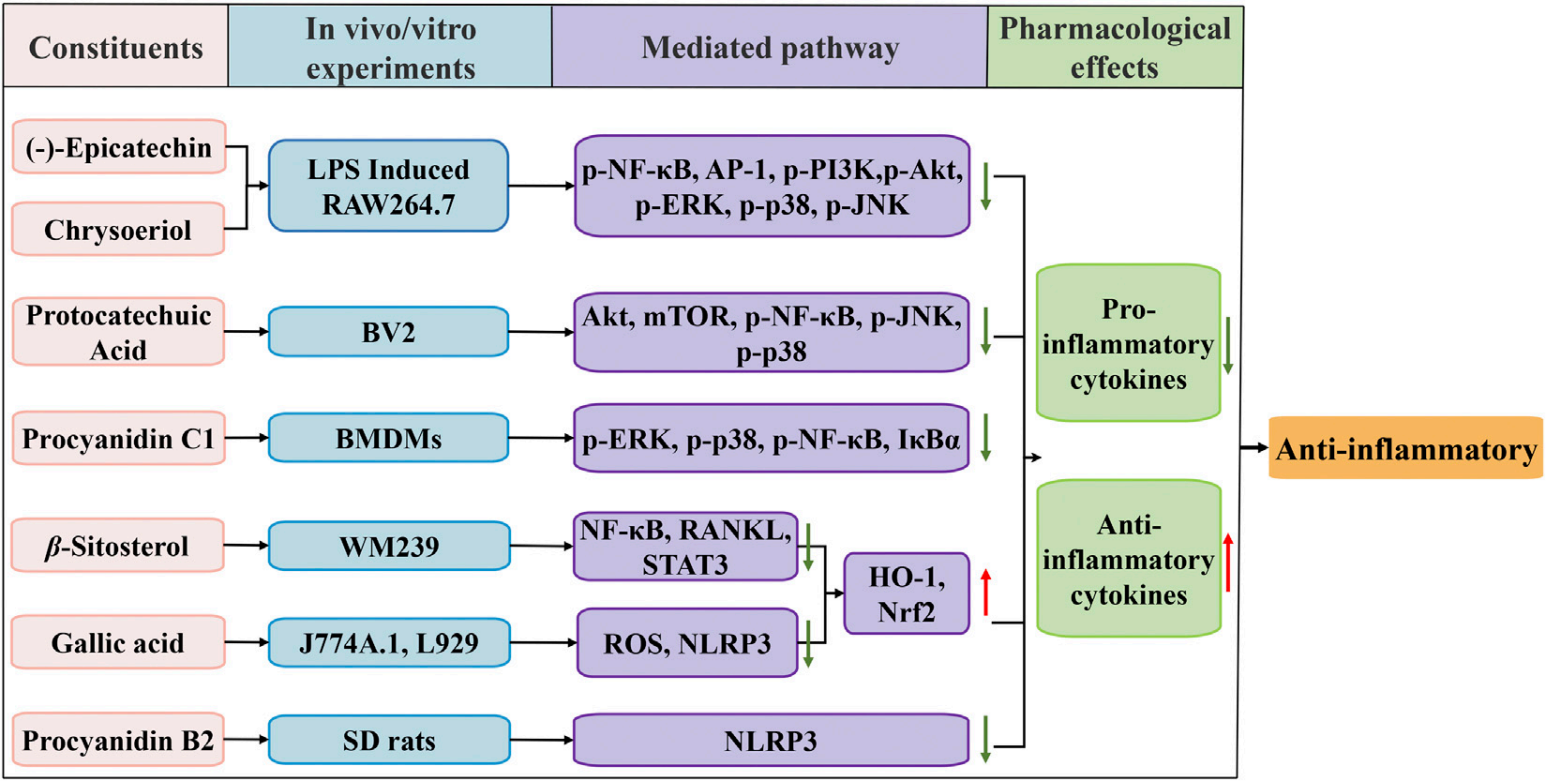
Schematic diagram of the anti-inflammatory mechanisms of FDR and its constituents.

Various *in vitro* and *in vivo* experiments investigated the anti-inflammatory effects of FDR extracts (Figure 3). The effects of (-)-epicatechin on lipopolysaccharide (LPS)-induced inflammation in RAW264.7 cells were demonstrated, and its anti-inflammatory effect may be related to a reduction in inflammatory cytokines, such as nitric oxide (NO), tumor necrosis factor-alpha (TNF-*α*), interleukin-1 and interleukin-6 (IL-6) and inhibition of the expression of nitric oxide synthase, phosphorylation of p38 mitogen-activated protein kinase (p-p38MAPK), extracellular signal regulated kinases 1/2 (ERK1/2) and c-Jun N-terminal kinase (JNK) (Ruan and Mu, 2017). Different (-)-epicatechin metabolites have anti-inflammatory properties that boost vascular health partially by reprogramming epigenetic signaling in endothelial-immune cells and reversing low-grade systemic inflammation (Milenkovic et al., 2020). Protocatechuic acid inhibited BV2 microglia and keratinocytes by reducing the activation of toll-like receptor 4 (TLR4)-dependent Akt, mTOR, and NF-*κ*B transcription factors and activating JNK and p38 MAPK (Wang H. Y et al., 2015; Amini et al., 2018; Nam and Lee, 2018). Rhamnetin treatment inhibited the inflammatory and proatherosclerosis pathways in ApoE−/− mice, and aortic tissue from ApoE−/−mice exhibited amelioration of TLR4 mRNA and components of the TLR4 pathway after treatment with rhamnetin (Wang et al., 2021).

By inhibiting the TLR4-mediated activation of NF-*κ*B and activator protein 1 and suppressing the phosphorylation of PI3K/Akt and MAPK, chrysoeriol inhibited the inflammatory response of LPS-stimulated RAW 264.7 cells (Yoon and Park, 2021). Gallic acid is a promising treatment for gouty arthritis. These effects are induced by suppression of ROS generation, which limits NOD-like receptor protein 3 (NLRP3) inflammasome activation and pyroptosis dependent on nuclear factor erythroid 2-related factor 2 (Nrf2) signaling (Lin et al., 2020). The anti-inflammatory properties of procyanidin b2 are attributed to suppression of NLRP3 inflammasome activation (Jiang et al., 2018). Byun et al. (Byun et al., 2013) indicated that procyanidin c1 inhibited LPS-induced activation of MAPK and NF-*κ*B signaling *via* TLR4 in macrophages. Zhang et al. (Zhang et al., 2020) demonstrated that *β*-sitosterol suppressed NF-*κ*B and activated heme oxygenase-1 (HO-1)/Nrf-2 pathways to inhibit arthritis.

FDR extract inhibits the transcription factor NF-*κ*B and the induced production of TNF-*α*, interleukin-8, IL-6, transforming growth factor-*β*1 and precollagen peptide III activity in chronic obstructive pulmonary disease rats, which improves lung tissue inflammation (Tang et al., 2014; Tang et al., 2016). The FDR extract prevented lung tissue injury in rats with pneumonia by downregulating TLR2/4, myeloid differentiation primary response 88 mRNA and NF-*κ*B inhibitor alpha protein expression (Dong et al., 2011). FDR tablets attenuated inflammatory symptoms and inflammatory damage in colorectal tissues of mice with a dextran sulfate sodium-induced inflammatory bowel disease model by downregulating TNF-*α*, IL-6 and interleukin-1*β* factor expression (Shen et al., 2019; Tan et al., 2020).

Clinical studies proposed combination therapy with Chinese medicines as an effective treatment strategy. FDR tablets combined with salazosulfapyridine (SASP) were more effective than SASP alone in ulcerative colitis (UC), and the mechanism may be the anti-inflammatory and immunomodulatory effects of intervening in UC *via* the TLR4/NLRP3 signaling pathway (Ge et al., 2021). FDR tablets were combined with compound kangfuxin solution and showed good efficacy in the treatment of UC (Hua and Yin, 2016). The effectiveness of FDR in controlling lung disease has been demonstrated in several clinical studies, including the treatment of adult and childhood bronchial asthma, and FDR capsules combined with salmeterol xinafoate and fluticasone propionate powder were effective (Li and Wu, 2018; Feng et al., 2021). Some studies also revealed that FDR tablets combined with cefoperazone and gubenkechuan tablets had a significant effect in chronic bronchial patients (Li, 2010; Han, 2020).

### 4.3 Immunomodulatory activity

Pharmacological studies confirmed that the extract from FDR showed an anti-rheumatoid arthritis effect, which may be due to its anti-inflammatory and immune activities (Shen, 2013). The polysaccharide content of FDR repairs the immune function of the thymus and spleen, enhances nonspecific immune function, improves specific humoral immunity and cellular immune function, and ultimately enhances the body’s immune function *via* multiple pathways, links, and targets (Gu et al., 2015). An extract of FDR reduced the expression of caspase-1, caspase-3, caspase-9, and matrix metallopeptidase-1 (MMP-1) in articular cartilage of a rabbit knee osteoarthritis model, which reduced cartilage damage and had an osteoprotective effect (Pan et al., 2019). FDR enhanced the phagocytosis of ventral and reticuloendothelial macrophages, which showed that it enhanced the immune function of mice (Yang et al., 1992; Zhang and Lin, 1999). Ethanol extract from FDR roots had an immunomodulatory role by promoting the proliferation of chicken spleen lymphocytes and the secretion of interleukin-2 and interferon-*γ* by peripheral blood T lymphocytes (Qiao et al., 2010).

### 4.4 Antioxidant activity

**FIGURE 4:**
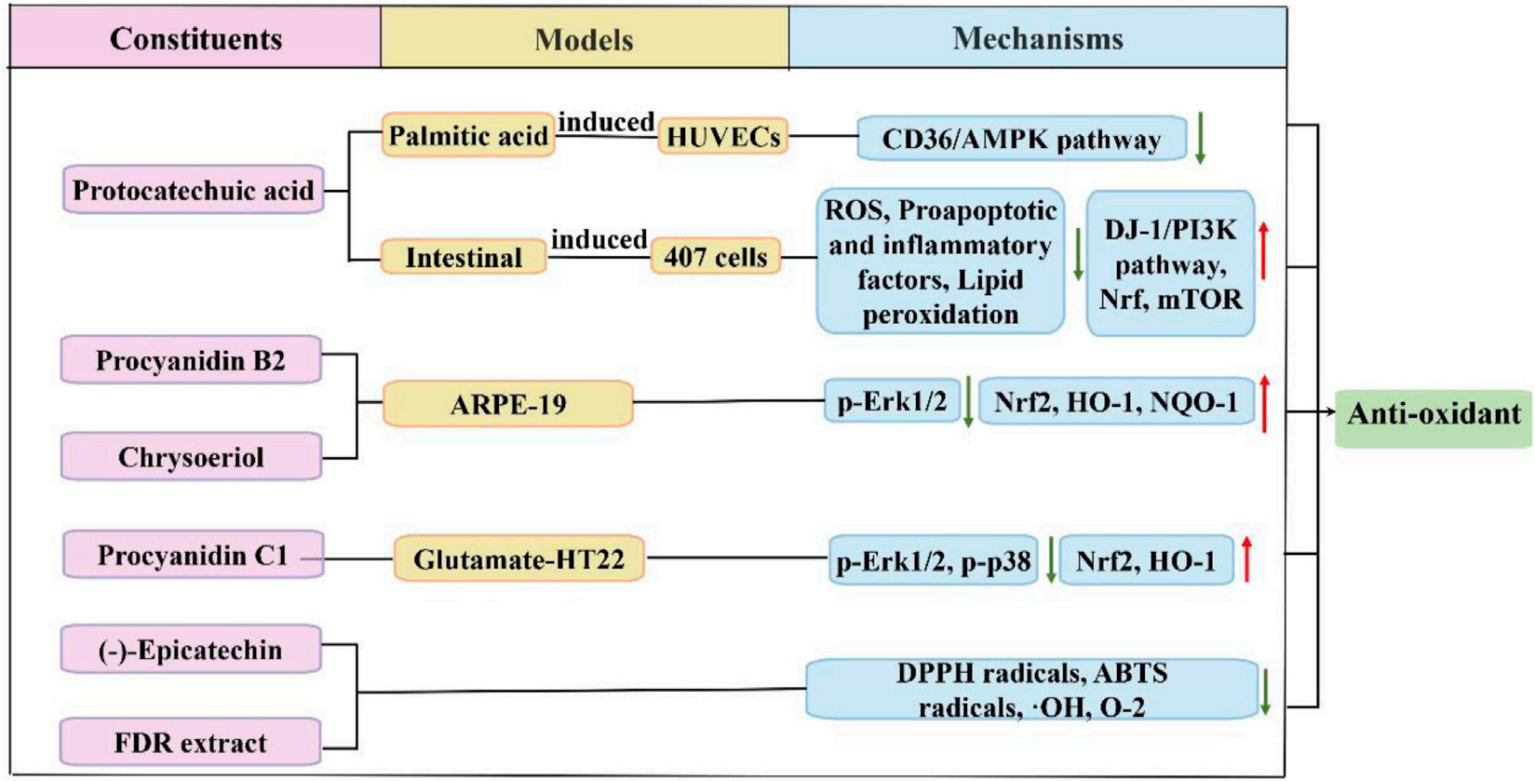
Schematic diagram of the antioxidant mechanisms of FDR and its constituents.

Organic acids, flavonoids, and tannins found in FDR demonstrate scavenging properties against free radicals and superoxide anions. Figure 4 shows the antioxidant effect of FDR *via* some pathways. Flavonoids remarkably reduced superoxide anion radicals and hydroxyl radicals in a concentration-dependent manner (Wang et al., 2017). Protoconuic acid is a naturally occurring organic acid that is widely distributed. Han et al. (Han et al., 2018; Han et al., 2019) found that the antioxidant properties of protocatechuic acid were beneficial for reducing the oxidative damage caused by palmitic acid in induced human umbilical vein endothelial cells (HUVECs) or high fat-induced oxidative damage in mice *via* downregulation of the CD36/AMPK-dependent pathway. PA had a beneficial effect on oxidative damage to the gastrointestinal mucosa by upregulating the DJ-1/PI3K pathways, increasing Nrf2 and mTOR expression, reducing ROS levels and lipid peroxidation, downregulating proapoptotic and inflammatory factors, and enhancing antioxidant enzyme activity and cell viability (Farombi et al., 2016; Cheng et al., 2019). (-)-Epicatechin in FDR extract exhibited stronger antioxidant activity and reduced superoxide anion radicals and hydroxyl radicals (Huang R et al., 2014). Procyanidin b2 prevented oxidative injury in aged mice *via* citrate cycle regulation, fatty acid regulation, and bile acid regulation, and procyanidin b2 suppressed intracellular ROS generation by activating Nrf2 expression to prevent oxidative damage (Xiao et al., 2018; Li B et al., 2021). Procyanidin c1 plays an important role in antioxidant activity by mediating the nuclear translocation of Nrf2 and increasing the expression levels of HO-1. Procyanidin c1 also blocks glutamate-induced phosphorylation of MAPKs, including ERK1/2 and p38, but not JNK (Song et al., 2019). Kim et al. (Kim et al., 2021) confirmed that chrysoeriol treatment prevented HO-induced oxidative stress in RPE cells, which significantly decreased the mitochondrial dysfunction caused by HO-induced oxidative stress. A reduction in MMP and an increase in mitochondrial-associated genes and proteins were also observed. Chrysoeriol also markedly induced the transcription factors Nrf2 and NAD(P)H:quinone oxidoreductase 1, which are related to antioxidants.

FDR leaf tea has significant antioxidant scavenging ability against DPPH radicals, ABTS radicals and hydroxyl radicals (Huang et al., 2016). FDR extract reduces cartilage damage by reducing malondialdehyde (MDA) and lipid peroxide content and enhancing superoxide dismutase (SOD) activity, which reduce oxygen free radicals and provide osteoprotective effects (Pan et al., 2020).

### 4.5 Antimicrobial activity

Ethanol extracts of FDR showed antimicrobial effects (Ai et al., 2002; Zhou et al., 2009). The ethanol extract of FDR showed inhibition of *β Hemolyticus Streptococcus* and *Streptococcus pneumoniae in vitro* and inhibited infections caused by strains of *Streptococcus pneumoniae* in mice *in vivo* (Yan et al., 2006). Fang et al. (Feng et al., 2006) evaluated the inhibitory activities of FDR against bacteria and fungi, and their results indicated that FDR exhibited obvious antibacterial effects on *Staphylococcus aureus*, *Escherichia coli* and *Diplococcus carriae* It also exhibited antifungal activities against *Giardia* and *Candida albicans*. FDR also demonstrated antibacterial activity by scavenging *Staphylococcus aureus, Bacillus subtilis* and *Saccharomyces cerevisiae* (Huang and Yi, 2015). KQH-01, KQH-02 and JQY-1 isolated from FDR showed strong antibiotic activity against some indicator microorganisms, such as *Staphylococcus aureus*, *Escherichia coli*, *Bacillus subtilis* and *Pythium aphanidermatum* (Zhang et al., 2011). FDR powder protected mice from *Salmonella* infection and suggested a dose-activity relationship (Wang et al., 2013).

FDR tablets combined with ceftriaxone had important antimicrobial activities in acute bacillary dysentery, and one of the mechanisms of action was the promotion of inflammatory absorption (Li, 2012). When FDR tablets combined with levofloxacin showed significant antimicrobial activity and may be used for the treatment of acute bacillary dysentery (Bi et al., 2012; Yu, 2014).

### 4.6 Antidiabetic activity

FDR flavonoids improved objective indices in streptozotocin-induced diabetes mellitus type 2 (T2DM) mice and regulated lipid metabolism and oxidative stress levels in model animals (Ruan et al., 2017). FDR leaf tea reduced blood glucose, blood lipids and MDA activity, increased SOD activity and improved pancreatic and liver lesions in mice with T2DM (Huang X et al., 2014). The FDR mixture significantly improved the clinical symptoms of diabetic nephropathy and significantly reduced the patient’s blood glucose, glycosylated hemoglobin and blood lipids (Huang et al., 2009).

### 4.7 Others

FDR also performs other functions in the above-described pharmacological activities. For example, FDR extract has an obvious antiviral effect *in vitro*, and its active ingredient is the flavonoid of FDR, which is concentration dependent (Zhao et al., 2019). Lianhua Qingwen associated with FDR tablets was more effective, faster and safer than oseltamivir alone in the treatment of patients with influenza A (Guo, 2015). Procyanidin b1 may be an effective treatment for hepatitis C virus, which may be an HCV RNA polymerase inhibitor (Li et al., 2010).

The tannic compound procyanidin b2 has analgesic effects, primarily *via* anti-inflammatory antioxidant free radicals to protect nerve cell membranes and prevent the production and release of the neurotransmitter 5-HT. It also antagonizes the ligand-type receptor 5-HT3A expression or promotes the expression of the G protein-coupled superfamily 5-HT1A receptor *via* the upstream signaling pathway to improve irritable bowel syndrome (IBS). Downregulation of transient receptor potential vanilloid 1 (TRPV1) expression also had a therapeutic effect on hyperalgesia in IBS rats (Liu et al., 2012a; Liu et al., 2012b; Liu et al., 2016). The hot plate test and the acetic acid twist test showed that FDR medicinal liquid had analgesic effects, and it increased the pain threshold and reduced the number of twists in mice (Pan and Wan, 2015). Jia et al*.* used dysmenorrhea models in mice to evaluate the analgesic effect of FDR extract and found that it showed potential analgesic activity (Jia et al., 2010).

Othman et al. suggested that the vasorelaxant effect of ethyl cinnamate was mediated *via* multiple pathways, and the inhibition of Ca^2+^ influx into vascular cells and release of NO and prostacyclin from endothelial cells were involved (Othman et al., 2002).

## 5 Conclusions and perspectives

The current review systematically discussed the ethnobotany, phytochemistry, and pharmacology of FDR. Various ailments have traditionally been treated with FDR, including chronic bronchitis, tumor, sore throat, rheumatic disease, dysentery, and enteritis. The predominant natural compounds in FDR are organic acids, tannins, and flavonoids, but over 100 compounds have been identified. FDR exerts antitumor, anti-inflammatory, immunomodulatory, antioxidant, antimicrobial, antidiabetic, and other pharmacological activities. In addition to the phytochemical and pharmacological studies mentioned above, FDR has also received considerable attention because it contains a variety of essential nutrients, and the chemical composition of human health has received widespread attention. Therefore, a better understanding of the phytochemistry and pharmacology of FDR will undoubtedly promote a more rational development and utilization of FDR.

FDR has rich nutritional value and healthcare functions. It is a medicinal resource plant with high developmental value. It contains organic acids, tannins, flavonoids, and other antitumor active ingredients, and gallic acid, procyanidin B2, (-)-epicatechin and genkwanin show significant antitumor activity. However, whether the antitumor effects of FDR are the result of the joint action of various components and the specific antitumor mechanism are not clear. Therefore, there is a need for in-depth research on the following aspects. According to pharmacodynamic studies, the effective site of the antitumor effect of FDR must be clarified *via* separation and purification to improve its antitumor potency. From the molecular or genetic level, more in-depth research is needed to reveal the antitumor effect of FDR. Because FDR has certain anti-invasive and metastatic effects, it is necessary to perform further research to understand the value and significance of its intervention in tumor cell invasion and metastasis. FDR tablets have also been clinically proven to increase the efficacy of pneumonia treatment, pulmonary abscess treatment, and rheumatic disease treatment. However, as a “Chinese Pharmacopoeia” collection, its mechanism of action of “removing heat and toxins and removing pus and stasis” must be further clarified. This pharmacological effect of FDR may be due to the extract or active ingredients as clearing and detoxifying lung agents in drinks and beverages to promote the full use of FDR resources. The antitumor and anti-inflammatory effects and mechanisms of FDR have been widely studied. However, the mechanism of antimicrobial, antidiabetic and immunomodulatory activity of FDR still needs to be further explored. The research on the mechanism of action of FDR should be continuously strengthened, and the potential medicinal function of it should be expanded to promote the development and utilization of its medicinal resources.
